# Assessing sexual health status among Tunisian ICU healthcare providers in times of the COVID-19

**DOI:** 10.1192/j.eurpsy.2022.1342

**Published:** 2022-09-01

**Authors:** A. Loghmari, M. Kahloul, K. Bouassida, Y. Slama, A. Harzali, M. Friaa, M. Soussi, M. Jaidane, W. Naija

**Affiliations:** 1 University hospital center of Sahloul, Urology Department, Sousse, Tunisia; 2 Sahloul Academic Hospital, University of medicine, “Ibn Al Jazzar”, Sousse, Tunisia, Department Of Anesthesia And Intensive Care, Sousse, Tunisia

**Keywords:** ICU healthcare providers, sexuality, Sexual health, covid 19

## Abstract

**Introduction:**

The COVID-19 pandemic has dramatically affected ones well-being. ICU healthcare providers are particularly concerned by this impact which includes physical, mental and socioeconomic repercussions. Others health dimensions could be deeply affected but not well explored such as the psycho-sexual status.

**Objectives:**

The aim of this study was to assess sexual health status among ICU healthcare providers.

**Methods:**

This was a cross-sectional study enrolling Tunisian ICU healthcare providers and conducted between July and September 2021. Data collection was based on a self-administrated questionnaire. To assess sexuality, Arabic validated versions of the IIEF-15 and the FSFI was used for male and female respectively. The Fear of COVID-19 Scale and the Rosenberg Self-esteem questionnaire were also used.

**Results:**

Twenty ICU workers (13 physicians and 7 nurses) were enrolled. The mean age was 28.2 years and the sex ratio was 2.3. All participants were involved in COVID-19 crisis management and 80 % reported an increase in their workload. The mean Rosenberg scale was 27 suggesting a low self-esteem. The mean Covid19 Fear Scale was 26 ± 2. For the IIEF-15 the mean score was 17 ± 3 (moderate erectyl dysfunction) and the most damaged dimension was the intercourse satisfaction. For the FSFI scale, the mean was 23 ± 5 witch (a low sexual dysfunction). A high sexual desire with a lack in the satisfaction dimension was reported in 90% of cases. Only 4 participants have consulted a sexologist.

**Conclusions:**

COVID-19 has a serious sexual impact in ICU healthcare providers justifying urgent psychological interventions.

**Disclosure:**

No significant relationships.

